# An unusual case of reactivated latent pulmonary cryptococcal infection in a patient after short‐term steroid and azathioprine therapy: a case report

**DOI:** 10.1186/s12890-021-01444-3

**Published:** 2021-03-04

**Authors:** Wei-Gang Pan, Bao-Chung Chen, Yao-Feng Li, Rui-Xin Wu, Ching-Hsun Wang

**Affiliations:** 1 Department of General Medicine, Tri-Service General Hospital, National Defense Medical Center, Taipei, Taiwan; 2 Division of Gastroenterology, Department of Internal Medicine, Tri-Service General Hospital, National Defense Medical Center, Taipei, Taiwan; 3 Department of Pathology, Tri-Service General Hospital, National Defense Medical Center, Taipei, Taiwan; 4 Division of Infectious Diseases, Tri-Service General Hospital Penghu Branch, Penghu, Taiwan; 5 Division of Infectious Diseases and Tropical Medicine, Department of Internal Medicine, Tri-Service General Hospital, National Defense Medical Center, No. 325, Section 2, Cheng-Kung Road, Neihu 114, Taipei, Taiwan

**Keywords:** Latent, Pulmonary cryptococcal infection, Steroid therapy, Azathioprine, Case report

## Abstract

**Background:**

*Cryptococcus* is one of the major fungal pathogens infecting the lungs. Pulmonary cryptococcal infection is generally considered a community-acquired condition caused by inhalation of dust contaminated with fungal cells from the environment. Here, we report a case developing pulmonary cryptococcosis 3 months after hospital admission, which has rarely been reported before.

**Case presentation:**

A 73-year-old female patient who was previously immunocompetent experienced persistent dry cough for 2 weeks, 3 months after admission. Chest computed tomography (CT) showed a new solitary pulmonary nodule developed in the upper lobe of the left lung. Staining and culture of expectorated sputum smears were negative for bacteria, acid-fast bacilli, or fungus. The patient then underwent biopsy of the lesion. Histopathology findings and a positive serum cryptococcal antigen titer (1:8) indicated pulmonary cryptococcosis. Daily intravenous 400 mg fluconazole was administered initially followed by oral fluconazole therapy. Follow-up chest CT after 3 months of antifungal therapy showed complete disappearance of the pulmonary nodule. Respiratory symptoms of the patient also resolved. A complete investigation excluded the possibility of a patient-to-patient transmission or primarily acquiring the infection from the hospital environment. Based on the patient’s history of exposure to pigeons before admission and recent steroid and azathioprine use after admission for the treatment of myasthenic crisis, reactivation of a latent pulmonary cryptococcal infection acquired before admission, in this case, is impressed.

**Conclusions:**

Although rarely reported, pulmonary cryptococcal infection should be included in the differential diagnosis of hospitalized patients with respiratory symptoms, especially in those with predisposing risk factors. Chest image studies and further surgical biopsy are needed for confirmation.

## Background

Basidiomycetous fungi of the genus *Cryptococcus* are ubiquitously present in the environment worldwide. Of these, *Cryptococcus neoformans* and *Cryptococcus gattii* are the two main species causing disease in humans [1]. Most infected cases are community-acquired, through inhalation of infectious fungal cells from the environment, but some occasionally reported cases are related to direct traumatic inoculation or mother-to-child transmission [2, 3]. Cases of hospital-acquired cryptococcal infections are quite rare. Herein, we present a case of prolonged hospitalization which developed pulmonary infection by *Cryptococcus neoformans* after short-term steroid and azathioprine use, that was possibly related to reactivation of endogenous pulmonary foci.

## Case presentation

A 73-year-old female patient, who was hospitalized since 3 months, presented with complaints of paroxysmal dry cough since 2 weeks. The patient was admitted initially due to progressive dyspnea. Respiratory distress occurred 2 days later and she was transferred to the intensive care unit (ICU) after intubation for mechanical ventilation. Her past medical history included hypertension and diabetes mellitus for approximately 20 years; both of which were in a stable condition with regular medication. Six months before admission, she underwent a median sternotomy and thymectomy for thymoma. She denied having a recent travel history aboard but reported contact with a large pigeon population as there was a large dovecot near her residence. A series of examinations were performed for the unexplained respiratory failure, and myasthenic crisis was diagnosed based on high levels of acetylcholine receptor antibodies (> 4 nmol/L) and neurophysiological study findings. Consequently, she received several courses of plasma exchange followed by a high dose of prednisolone (30 mg daily) and azathioprine (25 mg daily) to treat the myasthenic crisis. Her respiratory condition improved after the therapy. She was transferred to the general ward after successfully weaning off mechanical ventilation. Oral prednisolone and azathioprine therapy was continued. After receiving prednisolone and azathioprine therapy for nearly 3 months, she developed dry cough. Physical examination revealed that respiratory rate was 15 breaths/min, blood pressure was 140/88 mmHg, heart rate was 80 beats/min, and body temperature was in the normal range. The remainder of the examination including cardiovascular, respiratory, and other organ systems was unremarkable. Laboratory evaluation included a peripheral white blood cell count of 8870/µL; hemoglobin, 10.8 g/dL; and platelet count of 299,000/µL. Her serum alanine aminotransferase level was 9 U/L; aspartate aminotransferase level was 19 U/L; blood urea nitrogen level was 13 mg/dL; creatinine level was 0.3 mg/dL; level of procalcitonin was 0.1 ng/Ml, and level of C-reactive protein was 1.78 mg/dL. Arterial blood gas analysis (with the patient breathing ambient air) revealed pH 7.47, PaCO_2_ 56.9 mmHg, PaO_2_ 66.7 mmHg, and oxygen saturation 99%.

**Fig. 1:**
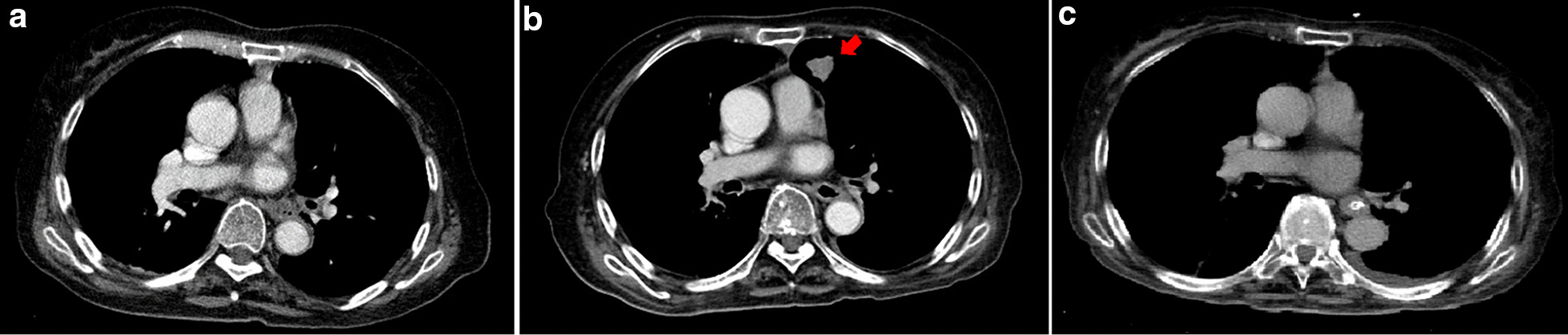
CT of the chest of nearly the same sections, at different times. CT performed **a** at admission in the emergency department revealed no active lung lesions. **b** At 3 months after admission, a newly developed solid nodule in the upper lobe of the left lung (red arrow) was observed. **c** At 3 months of fluconazole therapy, complete clearance of the lesion is observed

**Fig. 2:**
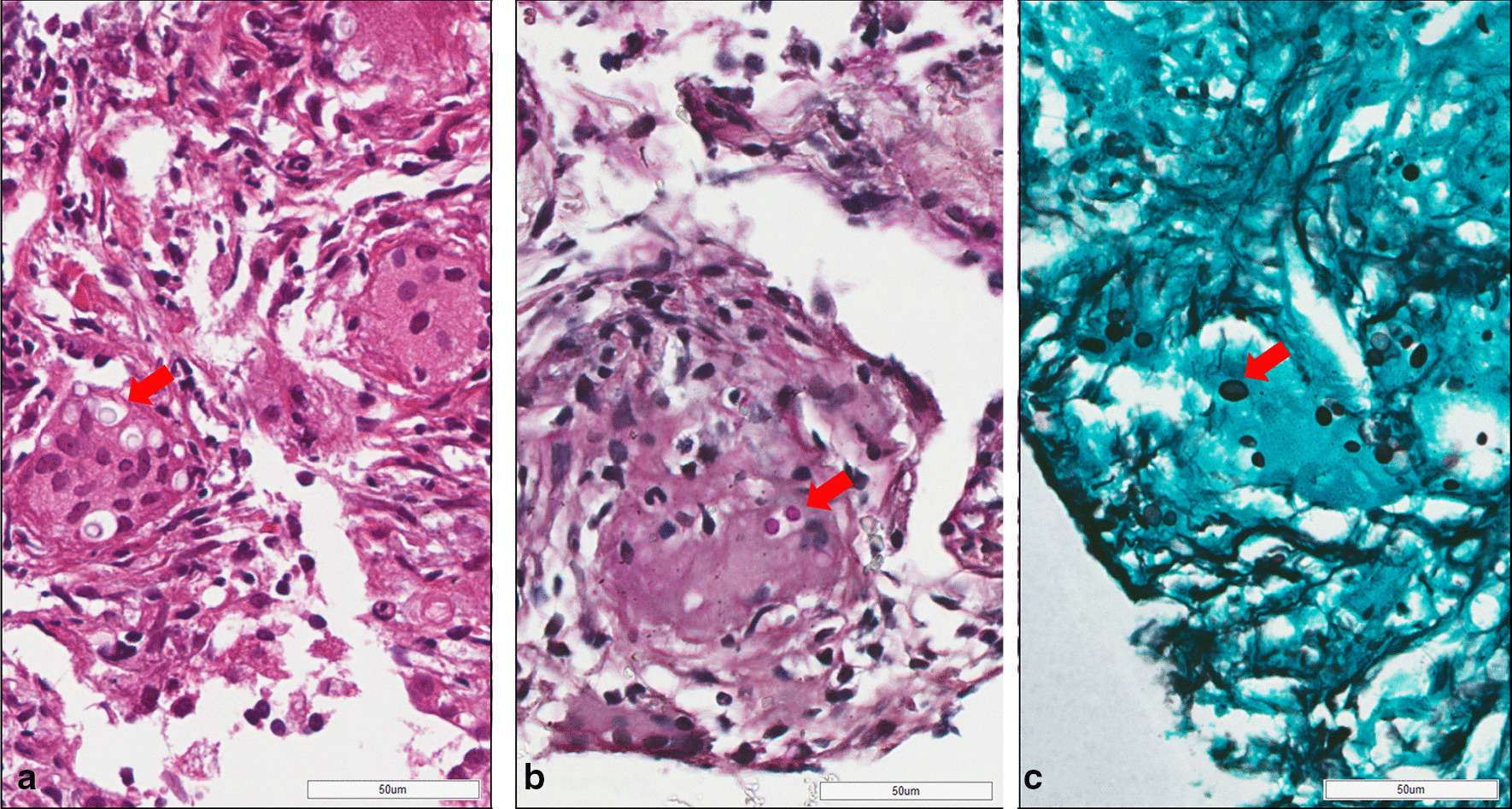
Histological examination revealed the presence of *Cryptococcus* (red arrow, 40 ×) with **a** hematoxylin and eosin stain, **b** periodic acid Schiff stain and **c** Grocott’s methenamine silver stain. These images were obtained from Digital Whole Slide Scanner, Aperio AT2. The scale bar is 50 µm

A thoracic CT scan was performed. On comparing it with the previous thoracic CT scan performed on admission (Fig. 1a), a newly developed 2-cm consolidative nodule was observed on the upper lobe of the left lung (Fig. 1b). Serum aspergillus galactomannan antigen testing, fungus stain and fungus culture of sputum, polymerase chain reaction (PCR) of sputum for mycobacterium tuberculosis were all negative. The CT-guided biopsy was performed, and its pathology revealed chronic granulomatous inflammation with multinucleated giant cells engulfing thick-wall micro-organisms (Fig. 2a). These organisms were 4–6 microns in diameter, stains bright red with Periodic Acid Schiff (Fig. 2b) and black in Gomori Methenamine-Silver (Fig. 2c), consistent with those of cryptococcus. A subsequent serum titer of cryptococcal antigen was 1:8 using Remel Cryptococcus Antigen Test Kit (Remel, Lenexa, KS, USA). Because there were no neurologic symptoms and signs indicated cryptococcus meningoencephalitis, lumbar puncture was not performed. Immune status of the patient was then assessed. Except for recent immunosuppressive therapy for the myasthenic crisis, the patient revealed no evidence of malignancy or vasculitis, and human immunodeficiency virus testing was negative. To determine the possibility of cross transmission from infected patients, we reviewed the incidence of cryptococcal infection cases in the ICU and the general ward for up to 1 month before the patient was diagnosed, but no new cryptococcal infection cases were reported. Intravenous fluconazole, 400 mg daily, was administered for pulmonary cryptococcal infection for 14 days. Respiratory symptoms nearly resolved after treatment and the patient was discharged for outpatient follow-up with continuation of oral fluconazole 400 mg daily. The chest CT scan, 3 months after discharge, revealed complete clearance of the lesion (Fig. 1c) and the relevant inflammatory biomarkers were in the normal range. No recurrence of the Cryptococcal infection has occurred to date.

## Discussion and conclusions

The incidence of invasive fungal infections has increased in the hospital setting in recent years. The reason may be the extensive use of immune system-suppressing modalities in patients, including stem cell transplantation, organ transplantation, chemotherapy, and immunosuppressive drug therapy. Such immunosuppressive therapies compromise the host immune system and make them vulnerable to invasive fungal infections [4]. The most common fungal species causing invasive disease is *Candida*, followed by *Aspergillus*. Cases due to other species such as *Mucorales* and *Fusarium* and other molds are less common [5]. For pulmonary invasive fungal infection, *Aspergillus* is the major pathogen. *Cryptococcal* infections are usually community-acquired, through inhalation of fungal cells from the environment, and rarely cause pulmonary infection in hospitalized patients. In our presented case, a comparison between the initial chest CT in the emergency department and that performed 3 months after admission (Fig. 1a, b) and the final pathology reports indicated pulmonary cryptococcal infection. This result surprised us, and an investigation was conducted to determine the possible infection source. We reviewed microbiology laboratory records in our hospital and found that there were no reported cryptococcal infection cases in the ICU and the general ward up to 1 month before our case was diagnosed of cryptococcal infection. This result excluded the possibility of a cluster outbreak from patient-to-patient transmission, which has been reported [6]. Additionally, during the prolonged hospitalization in the ICU and the general ward, the patient always stayed in bed, in an environment under a central air-conditioning system without open windows. This excluded the occurrence of acute primary infection through inhalation of fungal cells from the outdoor environment during hospitalization. Environmental sampling for this case, however, was not performed. Therefore, we could not completely exclude the possibility that our case acquired the infection from the hospital environment as reported before [7, 8]. Based on her contact history, we speculated that the pulmonary cryptococcal infection may be a result of a reactivated endogenous latent infection acquired before admission, which was precipitated by the recent immunosuppressant therapy. In immunocompetent hosts, primary pulmonary cryptococcal infections are cleared by the immune system or they establish themselves as latent infections within the thoracic lymph nodes or pulmonary granuloma for a prolonged period. On subsequent immunosuppression, *Cryptococcus* can reactivate and then disseminate to other tissues, thereby causing a life-threatening invasive disease such as cryptococcal meningoencephalitis [9, 10]. Our presented case showed only mild respiratory symptoms without neurological symptoms at disease onset, which rapidly improved after fluconazole use. Lumbar puncture, therefore, was not performed. Hospital-acquired (48 h or more after admission) pulmonary cryptococcal infections are very rare, with only three cases found in a literature review (Table 1) [6, 11, 12]. All reported cases had chronic lung disease, which has been reported as a risk factor for cryptococcal infection [13]. Although our present case had no underlying disease risk for cryptococcal infection, steroid and azathioprine use after admission for the myasthenic crisis may have decreased the immune defense and contributed to subsequent reactivation of latent pulmonary cryptococcal infection. Glucocorticoids can affect the pathogen-clearing function of various phagocytic cells including neutrophils, monocytes, and macrophages, which play important roles in host defense against cryptococcal infection [14]. Steroid usage, therefore, has been associated with subsequent cryptococcal infection, irrespective of short- or long-term use [12, 15, 16]. Azathioprine, which inhibits purine synthesis along with B and T lymphocytic cells function, can also compromise the patients’ immune system and make them vulnerable to opportunistic infections [17].

**Table 1:** Summary of previous reports of hospital acquired pulmonary cryptococcal neoformans infection cases

Patient no.	Age, year/sex	No. of days from admission to confirmed cryptococcosis	Underlying condition	Previous immunosuppressant use	Samples that had *Cryptococcus* growth	Outcome	References
1	73/female	41	Diabetes mellitus and hypertension	Steroid and azathioprine	Lung	Survived	Present case
2	82/male	4	Coronary artery disease and emphysema	Steroid	Lung	Death	[12]
3	63/male	58	Coronary artery disease and chronic obstructive disease	None	Blood, lung	Death	[11]
4	80/male	44	Lung cancer	Unknown	Lung	Death	[6]

The major limitations of this investigation were the lack of environmental sampling and direct culture of *Cryptococcus neoformans*. However, based upon pathology findings, clinical history and epidemiologic investigation results, we speculate that our presented case of pulmonary *Cryptococcosis* may be due to reactivated latent infection. In the evaluation of inpatients receiving immunosuppressive treatments with infection, reactivated latent infection such as pulmonary cryptococcosis should not be missed.

## Data Availability

All the data and materials shown in this report are acquired from the authors on reasonable request.

## Abbreviations

CT: Computed tomography
ICU: Intensive care unit
PCR: Polymerase chain reaction
